# Overexpression of Activated AMPK in the *Anopheles stephensi* Midgut Impacts Mosquito Metabolism, Reproduction and *Plasmodium* Resistance

**DOI:** 10.3390/genes12010119

**Published:** 2021-01-19

**Authors:** Chioma Oringanje, Lillian R. Delacruz, Yunan Han, Shirley Luckhart, Michael A. Riehle

**Affiliations:** 1 Advanced Testing Laboratory, Cincinnati, OH 45242, USA; chyoma12@yahoo.com; 2 Department of Entomology, University of Arizona, Tucson, AZ 85721, USA; lrdelacruz@email.arizona.edu; 3 Department of Health Sciences, ECPI University, Virginia Beach, VA 23462, USA; yunanhan4@gmail.com; 4 Department of Entomology, Plant Pathology and Nematology, University of Idaho, Moscow, ID 83844, USA; sluckhart@uidaho.edu; 5 Department of Biological Sciences, University of Idaho, Moscow, ID 83844, USA

**Keywords:** AMPK, *Anopheles stephensi*, midgut, *Plasmodium falciparum*, malaria, metabolism, reproduction

## Abstract

Mitochondrial integrity and homeostasis in the midgut are key factors controlling mosquito fitness and anti-pathogen resistance. Targeting genes that regulate mitochondrial dynamics represents a potential strategy for limiting mosquito-borne diseases. AMP-activated protein kinase (AMPK) is a key cellular energy sensor found in nearly all eukaryotic cells. When activated, AMPK inhibits anabolic pathways that consume ATP and activates catabolic processes that synthesize ATP. In this study, we overexpressed a truncated and constitutively active α-subunit of AMPK under the control of the midgut-specific carboxypeptidase promotor in the midgut of female *Anopheles stephensi*. As expected, AMPK overexpression in homozygous transgenic mosquitoes was associated with changes in nutrient storage and metabolism, decreasing glycogen levels at 24 h post-blood feeding when transgene expression was maximal, and concurrently increasing circulating trehalose at the same time point. When transgenic lines were challenged with *Plasmodium falciparum*, we observed a significant decrease in the prevalence and intensity of infection relative to wild type controls. Surprisingly, we did not observe a significant difference in the survival of adult mosquitoes fed either sugar only or both sugar and bloodmeals throughout adult life. This may be due to the limited period that the transgene was activated before homeostasis was restored. However, we did observe a significant decrease in egg production, suggesting that manipulation of AMPK activity in the mosquito midgut resulted in the re-allocation of resources away from egg production. In summary, this work identifies midgut AMPK activity as an important regulator of metabolism, reproduction, and innate immunity in *An. stephensi*, a highly invasive and important malaria vector species.

## 1. Introduction

AMP-activated protein kinase (AMPK), is a ubiquitous energy sensor and a major regulator of energy homeostasis in cells. It is a heterotrimer consisting of a catalytic alpha (α) subunit, a regulatory gamma (γ) subunit and a scaffolding beta (β) subunit. When cellular energy is reduced, AMPK detects increased intracellular AMP levels and promotes ATP production by switching off anabolic processes, such as glycogen, protein, and fatty acid synthesis, and activating catabolic processes, such as glycolysis and fatty acid oxidation [1]. AMPK activation leads to inhibition of glycogen synthase and activation of phosphofructokinase 2 (PFK2), reducing glycogen synthesis and increasing glycogenolysis, respectively. AMPK phosphorylates acetyl-CoA carboxylase 1 and 2 (ACC1/2), switching off fatty acid synthesis and switching on fatty acid oxidation respectively [1,2]. Furthermore, AMPK is a negative regulator of protein synthesis through its phosphorylation of several regulatory enzymes. These include (i) increased phosphorylation and subsequent inhibition of eukaryotic elongation factor 2 kinase (eEF-2K) leading to reduced translational processes [3,4], (ii) phosphorylation of tuberous sclerosis protein 2 (TSC2), inhibiting Rheb activity and decreasing activation of the mammalian target of rapamycin (mTOR) complex [5] and (iii) direct phosphorylation of Raptor, an mTOR binding partner, resulting in its binding to 14-3-3 proteins and excluding it from the mTOR complex [6].

Due to these metabolic roles, AMPK is an important regulator of several key physiologies, including lifespan, reproduction and mitochondrial biology. Among the most widely studied and vital roles is that of AMPK in the aging process, due to its regulation of several processes and pathways implicated in lifespan extension, including lipid metabolism, autophagy, mitochondrial health, TOR signaling and insulin signaling. Several studies linked calorie restriction-dependent lifespan extension to AMPK activation in the nematode *Caenorhabditis elegans* [7,8,9,10,11] and the fruit fly *Drosophila melanogaster* [12] and the promotion of healthy aging in mice [13]. In *C. elegans*, increased expression of the AMPK catalytic α2 subunit AAK-2 increased lifespan [9,10]. Lifespan of *D. melanogaster* was similarly extended by AMPK up-regulation in muscle, fat body, intestine and nervous system [12,14]. However, activation of *D. melanogaster* AMPK with dietary metformin failed to extend lifespan [15]. In *Aedes aegypti* mosquitoes, provision of polyphenol-rich diets, known to activate AMPK, enhanced *Ae. aegypti* lifespan [16], while similar diets had no effect on *Anopheles stephensi* lifespan [17]. 

AMPK regulates total energy stores and thus has an impact on nutrient intensive physiologies such as reproduction. In *C. elegans*, expression of a truncated, constitutively active AAK-2 led to reduced total egg production relative to wild type controls [18]. Interestingly, egg production was delayed in AAK-2 mutant worms and increased at later timepoints relative to controls, but this temporal change in egg production was not enough to overcome an overall reduction in fecundity. In rats, treatment with an AMPK activator led to reduced progesterone secretion [19]. In nutrient-restricted *D. melanogaster*, AMPK activity was necessary to slow the development of germline stem cells and ovarian follicle cells, while in well-fed flies, AMPK controlled germline stem cell maintenance and germline cyst formation [20]. In a similar fashion, nutrient-restricted *Georgecraigius atropalpus* mosquitoes exhibited delayed follicle development when provisioned with the AMPK activator 5-aminoimidazole-4-carboxamide riboside (AICAR) in a sugar meal [21].

In addition to its effects on lifespan and reproduction, AMPK is fundamental to mitochondrial function. AMPK maintains mitochondrial homeostasis by inducing mitochondrial biogenesis [22,23] through the phosphorylation of PGC1α [24] and mitophagy through phosphorylation of autophagy-related genes [25]. AMPK also regulates mitochondrial metabolism and dynamics via effects on catabolism of numerous substrates, sensing of metabolic cues, fusion, fission and transport [26,27]. Mitochondrial integrity and homeostasis in the midgut are closely linked to mosquito fitness and immunity [28,29,30]. In *An. stephensi*, an imbalance between mitochondrial biogenesis and autophagy was associated with increased production of mitochondrial nitric oxide (NO), which resulted in extreme resistance to *P. falciparum* infection, but a shortened lifespan [31].

In this study, we genetically engineered *An. stephensi* to express a constitutively active AMPKα subunit under the control of the midgut-specific carboxypeptidase (CP) promoter to examine the role of AMPK in mosquito metabolism and fitness. AMPK transgenic (TG) mosquitoes were significantly less likely to be infected with *P. falciparum*, exhibited changes in nutrient metabolism consistent with signaling of low energy reserves and had reduced reproductive output, suggesting that midgut AMPK activity in the midgut epithelium underlies life history tradeoffs for enhanced resistance to malaria parasite infection.

## 2. Materials and Methods

### 2.1. Mosquitoes

*An. stephensi* (Liston) mosquitoes were reared as described previously [32]. Briefly, mosquitoes were maintained at 27 °C and 70% humidity on a 16:8 h day:night cycle. Larval mosquitoes were fed ground cat food pellets (Purina; St. Louis, MO, USA). Adult mosquitoes were provided a 10% sucrose solution ad libitum. Human blood (American Red Cross, Washington DC; Institutional Biosafety Committee protocol 2010-014), used for colony maintenance and blood-feeding experiments, was warmed to 37 °C and provisioned to the mosquitoes via an artificial membrane feeder. During each generation, hemizygous mosquitoes engineered with a constitutively active AMPK α subunit were out-crossed with wild type colony mosquitoes to enhance genetic diversity and minimize fitness effects. This resulted in a 50:50 mix of hemizygous TG and non-transgenic (NTG) sibling mosquitoes reared under identical conditions. TG and NTG mosquitoes were separated at the pupal stage based on eye fluorescence using an Olympus SZX10 fluorescent stereomicroscope (Olympus, Tokyo, Japan) with enhanced green fluorescent protein (EGFP) filters. To establish a homozygous AMPKα line, hemizygous TG males and females from the same line were allowed to mate and then blood-fed. Immediately after eclosion, mating pairs of one F1 TG virgin female and one F1 TG male were established. To confirm that the resulting offspring were homozygous, ten individual F2 males from mating pairs that produced 100% EGFP-positive offspring were crossed with virgin NTG females. If all offspring expressed EGFP, the remaining F1 parental progeny were used to establish a homozygous line. For feeding experiments, engorged females were separated immediately after blood-feeding from unfed and partially fed mosquitoes and maintained on 10% sucrose. Females used for post-oviposition studies were transferred at 48 h post-bloodmeal (post-BM) to a new container and allowed to oviposit on moistened filter papers for 48–72 h.

### 2.2. Generation of TG An. stephensi Overexpressing AMPKαT176D under the Control of the Midgut- and Bloodmeal-Specific Carboxypeptidase Promoter 

The *Anopheles gambiae* carboxypeptidase (CP) promoter was modified to remove the signal peptide, start methionine and Kozak consensus sequence and ligated into the phsp-pBac shuttle plasmid containing a SV40 3’ UTR as described in Corby-Harris [32]. The kinase-encoding domain of the *An. stephensi* AMPKα catalytic subunit was amplified using species-specific primers (Table S1), then modified using site-directed mutagenesis to replace Threonine 176 (T176) in the activation site to Aspartic acid (Asp176) as described by Crute et al. [33]. The T176D mutation (Figure S1A–C) mimics the effect of phosphorylation and, therefore, enzyme activation. A similar construct used in other studies was resistant to protein phosphatases and was sufficient to maintain increased AMPK activity in tissues [34,35]. A NotI restriction site, Kozak consensus sequence (CCAACCATGG), and a human influenza hemagglutinin (HA)-epitope (YPYDVPDYA) were added to the N-terminus, while a SalI restriction site was added to the C-terminus to facilitate vector ligation, expression and protein detection respectively. The HA-AMPKαT176D construct was ligated into the modified pSLfa1180fa shuttle vector [36] containing the CP promoter and SV40 3’ UTR. Finally, the CP-HA-AMPKαT176D-SV40 construct was ligated into the pBac[3XP3-eGFPafm] vector [36] to generate the final construct, pBac[3XP3-eGPFafm]CP-HA-AMPKαT176D for injection into *An. stephensi* embryos by the University of Maryland Biotechnology Institute-Insect Transformation Facility (UMBI-ITF; Rockville, MD). All PCR amplified cDNAs, plasmids, and the final construct were sequenced to verify that construct sequences were correct. Transgene insertion sites in the mosquito genome were identified by inverse PCR [37]. Lifespan and reproduction studies were initiated only after outcrossing the TG lines to wild type mosquitoes for a minimum of three generations.

### 2.3. Transcript and Protein Expression Analysis of the CP-HA-AMPKαT176D Transgene

Midguts and carcasses (whole body minus midgut) were dissected from five 3–5 d old hemizygous TG and NTG sibling mosquitoes prior to blood-feeding (NBF) and at 6, 12, 24, 36, 48, and 72 h post-BM. Developmental expression of the transgene was assessed in 2nd and 4th instar larvae, pupae and 3–5 d old adult males and females. All samples were stored at −80 °C in RNAlater (Life Technologies, Grand Island, NY, USA) prior to RNA isolation or at −20 °C in 10X cOmplete^TM^ protease inhibitor/PhosStop^TM^ phosphatase inhibitor cocktail (Roche Diagnostics, Indianapolis, IN, USA) before protein isolation. Total RNA was extracted using the RNeasy Mini Kit (Qiagen, Germantown, MD, USA) and treated with DNase 1 (Fermentas, Thermo Scientific, West Palm Beach, FL, USA) to remove contaminating genomic DNA. cDNA was synthesized using the High-Capacity cDNA Reverse Transcription Kit (Applied Biosystems, Woburn, MA, USA) with random hexamers. The cDNA templates were subjected to RT-PCR amplification with GoTaq master mix (Promega, Madison, WI, USA) using primers complementary to the HA-epitope sequence and AMPKαT176D (Table S1) to detect the transgene in the midgut and carcass. To validate cDNA integrity and amplification conditions, cDNA samples were subjected to amplification with *An. stephensi*-specific actin primers (Table S1), while DNase-treated RNA samples served as no-template negative controls to verify a lack of genomic DNA and amplicon contamination. 

Immunoblot analyses were performed with one midgut equivalent of protein from a pool of five midguts as previously described with minor modification [32]. CP-HA-AMPKαT176D protein was detected using an anti-HA antibody (1:5000, Roche Applied Science; Penzberg, Germany). Phosphorylated endogenous *An. stephensi* AMPKα (p-AMPKα) was detected using an AMPKα antibody that recognizes phosphorylated Thr172 in humans, the phosphorylation site that is highly conserved with *An. stephensi* Thr176 (1:10,000; Cell Signaling Technologies, Danvers, MA, USA). Anti-glyceraldehyde 3-phosphate dehydrogenase (GAPDH) antibody (1:10,000; Cell Signaling Technologies, Danvers, MA, USA) was used as a loading control. Densitometry analysis of HA-AMPKαT176D or p-AMPKα relative to the GAPDH was performed with Image Studio Lite (LI-COR Biosciences, Lincoln, NE) or ImageJ (NIH). All experiments were replicated three times using independent cohorts of mosquitoes. Post-BM points were compared with NBF controls following normalization of the GAPDH controls among the three immunoblots and one-way ANOVA followed by a Dunnett’s multiple comparisons test. To verify the subcellular localization of CP-HA-AMPKαT176D, nuclei and cytoplasmic fractions were prepared from the midguts of homozygous TG *An. stephensi* as described by Brown et al. [38]. These fractions were subjected to immunoblot analysis using the anti-HA antibody as described above with replicated samples from three independent cohorts of mosquitoes.

### 2.4. Quantification of An. stephensi Macronutrients

To assess the effect of HA-AMPKαT176D on metabolism, we examined the levels of glycogen, trehalose and lipids in TG adult females and compared to NTG or wild type female mosquitoes. Whole-body homogenates of 3–5 d old female TG (homozygous or hemizygous), NTG and wild type *An. stephensi* were collected at various time points (NBF, 24 h, 48 h, and 72 h post-BM). Glycogen, lipids and trehalose were isolated using a procedure described by Van Handel [39] and modified by Zhou et al. [40] and Telang and Wells [41]. Fractions containing glycogen, lipid or trehalose were dried and kept at 4 °C until quantification by colorimetric-based assays. Total lipids were determined by modified Vanillin reagent assay [42] and total glycogen and trehalose by a modified anthrone-based assay [43]. Commercially available standards of triolein (Sigma-Aldrich, St. Louis, MA, USA), glycogen (Thermo Scientific; Waltham, MA, USA) and trehalose (Thermo Scientific; Waltham, MA, USA) were reconstituted at 1.0 mg/mL, from which aliquots were prepared to generate a standard calibration curve. Each sample was assayed in triplicate at appropriate wavelengths using a spectrophotometer (Multiskan Go by Thermo Scientific; Waltham, MA, USA), and technical replicates were averaged for each biological replicate. All nutrients are reported as micrograms per mosquito. Each experiment was replicated 13 times using independent cohorts of mosquitoes and TG versus NTG treatments were compared using the Student t-test. 

### 2.5. Lifespan Studies

Transgenic hemizygous CP-HA-AMPKαT176D female *An. stephensi* were mated with wild type mosquitoes to generate a 50:50 TG to NTG sibling ratio. Female mosquitoes were separated into four treatment groups: TG blood-fed, NTG blood-fed, TG sugar-fed, and NTG sugar-fed. Blood-fed mosquitoes were given daily bloodmeals in addition to 10% sucrose ad libitum and sugar-fed mosquitoes were maintained only on 10% sucrose ad libitum. Dead mosquitoes were counted and removed daily until all mosquitoes perished. Lifespan studies were also carried out on homozygous CP-HA-AMPKαT176D and wild type female controls. Each experiment was replicated 2–5 times with independent cohorts of mosquitoes (>90 females per independent cohort). Percent survival was calculated for each cohort and an average of all replicates was plotted. Survival curves were analyzed using the Kaplan-Meier method via statistical software JMP 13 (SAS Institute Inc., Cary, NC, USA.) and significant differences were detected using the Wilcoxon test. Curves were considered significantly different at *p* ≤ 0.05.

### 2.6. Reproduction Studies

Lifetime fecundity assays (total eggs laid by a cohort over their lifespan) were conducted in concert with the blood-fed lifespan studies above. To assess lifetime fecundity, oviposition substrates were placed into the cages of blood-fed groups (homozygous/WT or hemizygous TG/sibling NTG) at 48 h post-BM and replaced every 48 h until the final mosquito perished. For egg reproduction per individual mosquito, we followed procedures described by Arik et al. [44]. Briefly, adult female mosquitoes from homozygous/WT or hemizygous TG/NTG crosses were blood-fed. Fully engorged females were collected and individually housed. A similar procedure was carried out for control NTG and wild type mosquitoes. At 48 h post-BM, oviposition cups were provided for individually housed females and they were allowed to oviposit for 48 h (i.e., 48 to 96 h post-BM). Oviposition cups were removed and eggs were counted manually under a light microscope. For lifetime fecundity analyses, the number of eggs per oviposition paper were estimated using Image J 1.42 software (National Institutes of Health) following the protocol described in Mains et al. [45] with manual counting of a random selection of oviposition papers to validate automated egg counting. Fecundity experiments were replicated with 25–30 mosquitoes from 2–3 independent cohort of *An. stephensi*. For each replicate, mean egg counts were analyzed for significant differences among groups using a Wilcoxon test.

### 2.7. Pharmacological Activation of AMPK

To assess the effects of the AMPK activator AICAR on *An. stephensi* fitness and metabolism, newly emerged, wild type mosquitoes were fed *ad libitum* on 10% sucrose containing 2.5 mM AICAR (Selleck Chemical LLC., Houston, TX, USA) or 10% sucrose containing an equivalent volume of AICAR diluent (phosphate-buffered saline, PBS) as a control. These mosquitoes were used for analyses of lifespan, lifetime fecundity and a metabolism. For lifespan and lifetime fecundity analyses, mosquitoes were maintained on 2.5 mM AICAR-supplemented sucrose or PBS-supplemented sucrose and provided a daily bloodmeal until all mosquitoes perished. For metabolism assays, 3–5 d old female mosquitoes were fed food dye-colored 2.5 mM AICAR-supplemented sucrose or PBS-supplemented sucrose to facilitate identification of fed mosquitoes. Whole bodies of five mosquitoes from AICAR-treated and control mosquitoes were collected pre-feeding and at 5 h post-feeding (corresponding to the expected period of increased AMPK expression due to CP promoter activity) and stored at −80 °C for further analysis as described above. 

### 2.8. P. falciparum Studies

Cultures of *P. falciparum* NF54 were initiated at 1% parasitemia in 10% heat-inactivated human serum, and 6% washed human red blood cells (RBCs) in RPMI 1640 with HEPES (Gibco, Gaithersburg, MD, USA) and hypoxanthine. Stage V gametocytes were evident by day 15, and exflagellation was evaluated on the day before and the day of mosquito feeding. For our assays, 5 d old female TG (homozygous or hemizygous) and NTG siblings (hemizygous control) or wild type (homozygous control) *An. stephensi* were fed on a mature gametocyte culture diluted with human RBCs and heat-inactivated serum. On day 10, midguts from fully gravid females were dissected in PBS and stained with 1% mercurochrome in PBS to visualize *P. falciparum* oocysts. Total oocysts were counted for each midgut. The mean number of oocysts per midgut (infection intensity) and prevalence of infected mosquitoes (the proportion of mosquitoes with at least one midgut oocyst) were calculated from all dissected mosquitoes. The experiments were independently replicated with two cohorts of >50 mosquitoes. Infection data were first analyzed by ANOVA to determine that infections in control groups were not significantly different among replicates. No significant differences were evident, so data were pooled across replicates and distributions were compared with a Mann–Whitney test for group differences. Infection prevalence was analyzed by Fisher’s exact test to identify differences between the groups. Differences were considered significant at *p* < 0.05.

## 3. Results

### 3.1. Generation and Characterization of CP-HA-AMPKαT176D Transgenic An. stephensi

**Figure 1:**
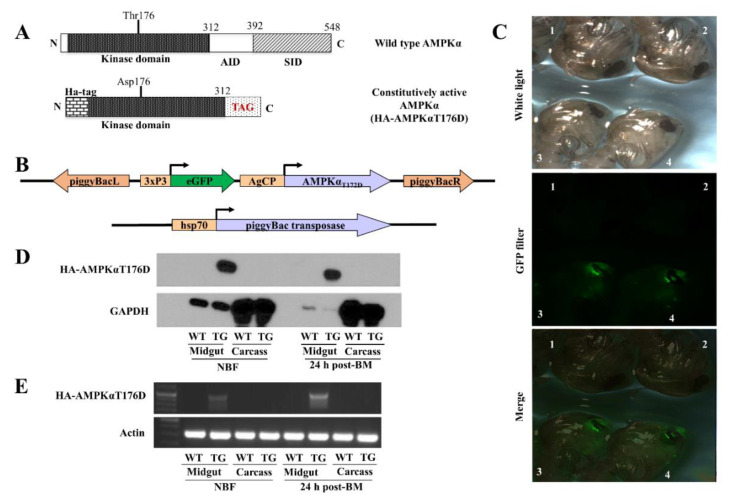
Generation of the HA-AMPKαT176D transgenic *An. stephensi* and protein and transcript expression profile of the transgene in adult females. (**A**) Full length AMPKα subunit versus constitutively active AMPKα subunit. Changes include removal of the inhibitory domains, conversion of Thr176 to Asp176 and the addition of an HA epitope. (**B**) Schematic of the construct genetically engineered into *An. stephensi* mosquitoes. (**C**) Comparison of transgenic (TG) and non-transgenic (NTG) siblings. Top panel: NTG (1 & 2) and TG pupae (3 and 4) under white light. Middle panel: TG and NTG under fluorescence and a GFP filter. Bottom panel: merge of top and middle panels. (**D**) Representative immunoblot of total proteins isolated from the midguts or carcasses (whole body minus midgut) of TG and NTG mosquitoes and probed with anti-HA antibody (top) or anti-GAPDH antibody (bottom) as loading control. (**E**) Total RNA was isolated from the midguts or carcasses of both TG and NTG mosquitoes and converted into cDNA. Transgene specific primers were used to amplify HA-AMPKαT176D (top). *Actin*-specific primers were used as positive control to verify the integrity of the cDNA (bottom). No template controls served as negative control. All transcript and protein expression studies were replicated a minimum of 3 times.

The AMPKαT176D mutation (Figure 1A) has been reported to mimic the effect of a phosphate group and eliminates the requirement for phosphorylation at the site, giving rise to a partially active enzyme that is resistant to protein phosphatases and is sufficient to maintain AMPK activity in tissue [33,34,35,46]. The construct was inserted into the pBac plasmid vector for transformation of *An. stephensi* genome (Figure 1B) by UMBI-ITF; successful transformation was detected by positive green eye fluorescence (Figure 1C). One EGFP-expressing TG line was generated and maintained as hemizygous by outcrossing every generation to wild type *An. stephensi* to maximize genetic diversity. Following several generations of outcrossing between hemizygous and wild type mosquitoes a homozygous line was also established.

**Figure 2:**
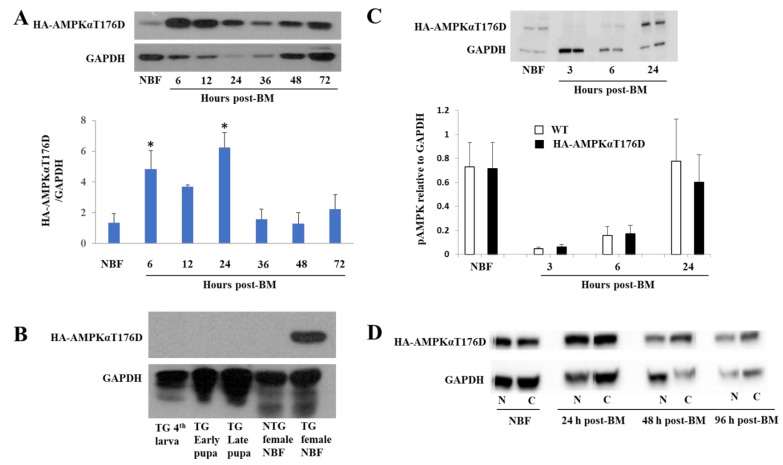
Expression profile of HA-AMPKαT176D. (**A**) Representative immunoblot detailing the expression of HA-AMPKαT176D and GAPDH in the midguts of non-blood-fed (NBF) and blood-fed TG mosquitoes at 6, 12, 24, 36, 48 and 72 h post-BM. The lower graph depicts the average expression of transgene protein normalized to GAPDH loading controls. Data are represented as means ± SEMs from three replicates with independent cohorts of mosquitoes. Significant differences between the post-BM treatments and the NBF controls were determining using a one-way ANOVA followed by a Dunnett’s multiple comparisons test (* *p* < 0.05). (**B**) HA-AMPKT176D protein expression at the different mosquito developmental stages. (**C**) Representative immunoblot of the expression of phospho-AMPK and GAPDH in the midguts of NBF and blood-fed (3, 6, and 24 h post-BM) WT and TG mosquitoes. Average expression of endogenous p-AMPK relative to GAPDH loading controls and shown relative to levels in NBF mosquitoes and in homozygous TG females compared to wild type at 3, 6, and 24 h post-BM. Data are represented as means from five replicates with independent cohorts of mosquitoes. (**D**) Three independent nuclear and cytoplasmic fractions of homozygous TG mosquito midguts collected at different time points post-BM were prepared and probed with anti-HA antibody and GAPDH as loading control.

We assessed HA-AMPKαT176D expression levels in different developmental stages and in midguts and carcasses of adult females prior to blood-feeding and at 24 h post-BM. In adult females, the transgene was expressed in a midgut specific manner (Figure 1D,E). No transcript or protein expression was observed in the carcass of TG mosquitoes or in the midgut or carcass of NTG mosquitoes. HA-AMPKαT176D protein was expressed at all the time points post-BM but increased protein expression was seen between 6 h and 24 h post-BM as expected for the CP promoter, after which the transgene level returned to pre-bloodmeal level (Figure 2A). HA-AMPKαT176D was detected only in adult TG females (Figure 2B). There was no effect of the transgene on endogenous p-AMPKα levels in the *An. stephensi* midgut at different time points post-BM. Specifically, while there were significant decreases in endogenous p-AMPKα levels at 3 h and 6 h post-BM followed by a return to pre-bloodmeal levels at 24 h (Figure 2C), these changes were observed in both TG and wild type mosquitoes.

### 3.2. Identification of Transgene Insertion Sites Using Inverse PCR

We utilized inverse PCR to identify the genomic insertion site of the CP-HA-AMPKαT176D construct in TG *An. stephensi* (Figure S1). BLAST analyses of the 5′ and 3′ ends of the inverse PCR amplicons allowed us to identify a single insertion site in the TG line that conformed to a TTAA sequence, the preferred site of piggyBac transposition [47]. Importantly, the sequences were not associated with any predicted gene in the *An. stephensi* genome and were not identified in any *An. stephensi* transcript databases. This suggests that the transgene was inserted into an intergenic region and, therefore, unlikely to induce fitness costs due to genic disruption caused by the transgene insertion.

### 3.3. Subcellular Localization of the HA-AMPKαT176D Protein

It has been reported that AMPKα subunits encode a highly conserved carboxyl-terminal tail with 22 amino acids that function as a nuclear export signal and for normal subcellular localization in vivo [48]. When designing our construct, we removed the autoinhibitory and subunit interacting domains containing these 22 amino acids as described for mammalian cell constructs [34,35]. Therefore, we sought to determine whether HA-AMPKαT176D localization was restricted to the nucleus due to the loss of these amino acids or whether movement into the cytoplasm occurred. We fractionated midgut tissues into nuclear, membrane and cytoplasmic fractions and compared HA-AMPKαT176D protein expression in these fractions. Both nuclear and cytoplasmic midgut fractions from TG *An. stephensi* had similar levels of HA-AMPKαT176D, indicating that the removal of these amino acids did not restrict our modified AMPKα subunit to the nucleus (Figure 2D).

### 3.4. Effect of HA-AMPKαT176D Expression on An. stephensi Nutrient Stores

**Figure 3:**
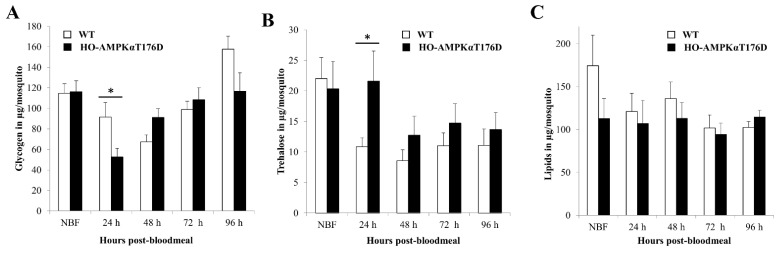
Effect of HA-AMPKαT176D overexpression on *An. stephensi* glycogen, lipids, and trehalose. (**A**) Glycogen (**B**) Trehalose, and (**C**) Lipids were extracted and assayed from pools of five females. Average concentrations ± SEMs are shown. Data were analyzed using Student’s *t* test (*n* = 15 biological replicates). * *p* < 0.05 relative to wild type controls.

We measured whole body titers of glycogen, trehalose and lipids in young (3–5 d old) hemizygous and homozygous TG mosquitoes relative to NTG and wild type control mosquitoes, respectively, at various time points post-BM. In contrast to a lack of change in lipid levels, we observed a significant decrease in glycogen and a corresponding significant increase in trehalose in homozygous TG mosquitoes relative to controls at 24 h post-BM when transgene expression was maximal (Figure 3A–C). Specifically, we observed a 43% decrease in glycogen and a 99% increase in trehalose (*p* < 0.05; Figure 3A,B). There were no observable effects on nutrient levels in hemizygous TG mosquitoes (Figure S2A–C), suggesting that gene dosage and expression levels may be too low in the hemizygous state to observe differences. We observed a similar reduction (28%) in glycogen levels in wild type mosquitoes at 5 h following AICAR administration (*p* = 0.05), but no corresponding increase in trehalose (Figure S3). AICAR has been reported to have differing effects in quiescent versus rapidly proliferating cells and its intracellular monophosphorylated nucleotide metabolite (ZMP) is much less potent than AMP as an AMPK activator [49], suggesting, as we have observed, that TG and AICAR-treated mosquitoes would exhibit similar but not identical biology. Collectively, these results are consistent with low energy conditions signaling by increased AMPK activity, which would prompt the mosquito to utilize stored energy—first glycogen, then lipids—to generate ATP and to increase circulating levels of trehalose.

### 3.5. Effect of HA-AMPKαT176D Expression on An. stephensi Lifespan

**Figure 4:**
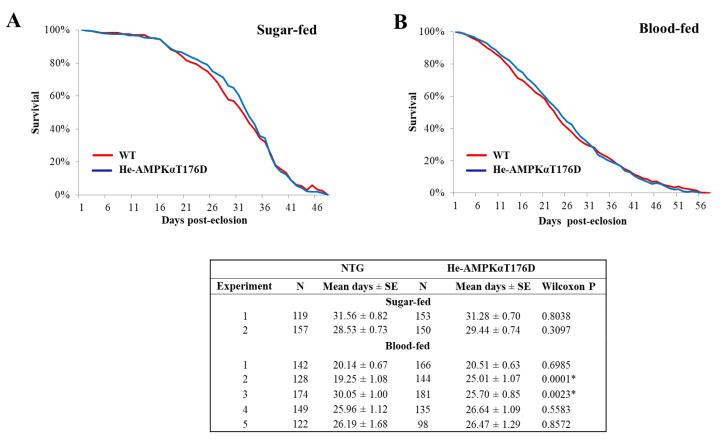
Survivorship of sugar-fed and blood-fed AMPKαT176D hemizygous transgenic (TG) and non-transgenic (NTG) *An. stephensi*. (**A**) A representative survivorship curve comparing hemizygous TG and NTG siblings reared under identical conditions and provided with 10% sucrose *ad libitum*. Lifespan experiments were replicated twice with independent cohorts of mosquitoes. (**B**) A representative survivorship curve comparing hemizygous TG and NTG siblings reared under identical conditions and provided with a daily bloodmeal and 10% sucrose solution. Lifespan experiments were replicated five times with independent cohorts of mosquitoes. The table summarizes the sample sizes, means, and statistical analyses results (* *p* < 0.05) of lifespan data using the Wilcoxon test for sugar-fed and blood-fed mosquitoes.

**Figure 5:**
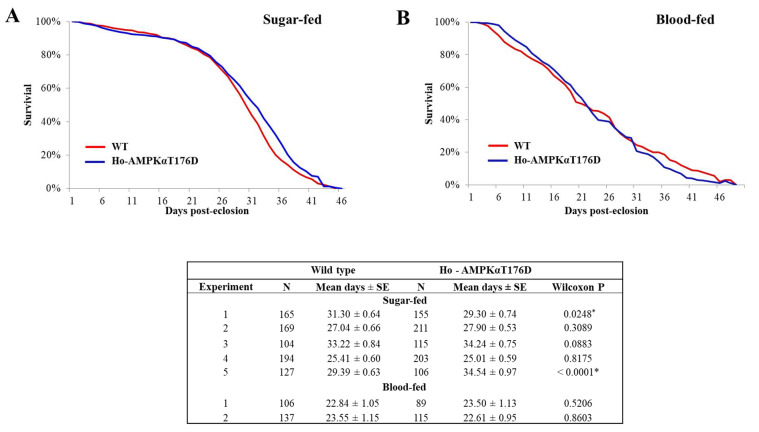
Survivorship of sugar-fed and blood-fed AMPKαT176D homozygous transgenic (TG) and wild type *An. stephensi*. (**A**) A representative survivorship curve comparing homozygous AMPKαT176D TG and wild type siblings reared under identical conditions and provided with 10% sucrose *ad libitum*. Lifespan experiments were replicated five times with independent cohorts of mosquitoes. (**B**) A representative survivorship curve comparing homozygous AMPKαT176D TG and wild type siblings reared under identical conditions and provided with a daily bloodmeal and 10% sucrose solution. Lifespan experiments were replicated twice with independent cohorts of mosquitoes. The table summarizes the sample sizes, means, and statistical analyses results (* *p* < 0.05) of lifespan data using the Wilcoxon test for sugar-fed and blood-fed mosquitoes.

Studies in *C. elegans* [9,10] and *D. melanogaster* [12,14] have shown that upregulation of AMPK improves survivorship. Further, the effect in *D. melanogaster* was observed when AMPK activity was increased specifically in the fly midgut [14]. Thus, we hypothesized that increased expression of HA-AMPKαT176D in the midgut would extend the lifespan of TG mosquitoes relative to NTG controls. Surprisingly, we observed no difference in survival between hemizygous TG and NTG mosquitoes, when maintained on 10% sucrose meal or when provided with daily bloodmeals, when survival curves were analyzed with the Wilcoxin signed rank test (Figure 4A,B and Figure S4). Sugar-fed hemizygous TG mosquitoes lived an average of 30.36 days (range 29.44–31.28) compared to 30.05 days (range 28.53–31.56) NTG siblings. A similar trend was observed when both groups of mosquitoes were provided a daily bloodmeal. Hemizygous TG lived an average of 24.87 days (range 20.51–26.64) compared to 24.32 days (range 19.25–30.05) in NTG siblings. Like hemizygous TG mosquitoes, the average lifespans of homozygous TG mosquitoes fed sugar or blood were not significantly different from the average lifespans of wild type *An. stephensi* fed sugar or blood. Although homozygous TG mosquitoes lived longer in the first replicate, the means of five replicates with sugar-fed mosquitoes were not different (30.20 versus 29.27 days in homozygous TG and wild type *An. stephensi*, respectively; Figure 5A and Figure S5). Neither replicate for blood-fed mosquitoes showed significant differences in survival curves (Figure 5B and Figure S5). An analysis of median lifespans in the seven sugar fed (one-way ANOVA, *p* = 0.66) and seven blood-fed (*p* = 0.84) cohorts of *An. stephensi* indicated that TG mosquitoes were not significantly different from matched controls. Similar to the lack of effect of HA-AMPKαT176D overexpression on *An. stephensi* lifespan, AICAR treatment had no effect on *An. stephensi* survival (Figure S6A).

### 3.6. Effect of HA-AMPKαT176D expression on An. stephensi Reproduction 

The metabolic effects of AMPK activation, including protein and lipid mobilization for energy generation [50,51], suggest that AMPK activation would impact energy- and resource-intensive reproduction. Further, given that decreases in macronutrients have been linked to delayed mosquito follicle development [21], we hypothesized that AMPK activation in *An. stephensi* would be associated with reduced reproduction. To test this hypothesis, we examined oviposition in TG and NTG sibling *An. stephensi* during individual gonotrophic cycles and over the course of lifetime egg production.

**Figure 6:**
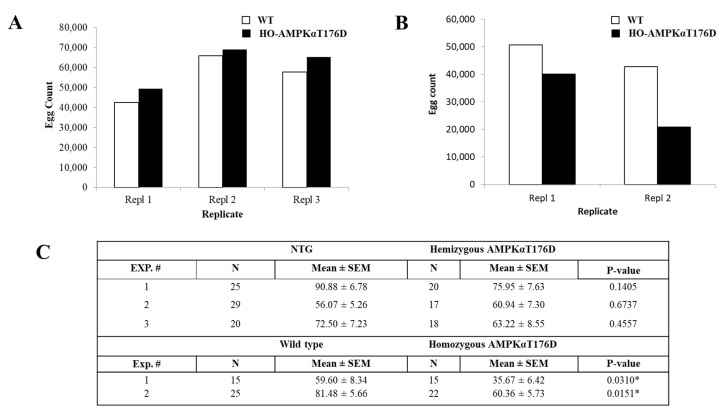
Impact of HA-AMPKαT176D overexpression on lifetime fecundity. Graphs represent total egg production of hemizygous (**A**) and homozygous (**B**) TG females compared with NTG or wild type control females, respectively, throughout their lifespans. No significant differences were observed between hemizygous TG and NTG or homozygous TG and wild type females (*p* > 0.05). (**C**) Egg counts for TG and wild type female mosquitoes. Results are presented for each replicate separately, including the number of females tested (N), the mean number of eggs laid ± SEMs, and *p*-values by Wilcoxon test.

Lifetime fecundity was not significantly different between hemizygous TG and NTG controls, although in two of three replicates hemizygous TG mosquitoes laid slightly more eggs (Figure 6A). To determine if these trends might result from differences in mosquito survival and, hence, extended duration of egg laying, we calculated the average number of eggs produced per surviving female at each gonotrophic cycle (total eggs produced divided by the number of surviving mosquitoes in the caged population). As with total population egg production, egg production per surviving female was not significantly different between hemizygous TG and NTG females (Figure S7A–C). In contrast, caged populations of homozygous TG females produced significantly fewer eggs than caged populations of wild type controls over their respective lifespans (*p* < 0.05, Figure 6B), and the average number of eggs produced per surviving female at each gonotrophic cycle was significantly lower in homozygous transgenic mosquitoes (Figure S7D,E). AICAR provisioning also significantly reduced egg production in wild type *An. stephensi* relative to controls (Figure S6B,C). As with the lifetime fecundity of caged populations, individual homozygous TG *An. stephensi* laid significantly fewer eggs than individual wild type controls (*p* < 0.05), but there were no differences in mean eggs produced by hemizygous TG mosquitoes compared to NTG sibling controls (Figure 6C).

### 3.7. Effect of HA-AMPKαT176D Overexpression on the Prevalence and Intensity of P. falciparum Infection in An. stephensi

**Figure 7:**
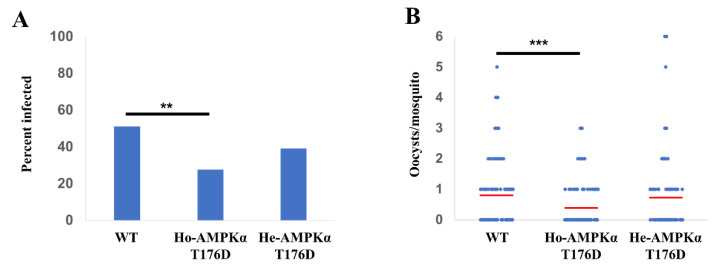
HA-AMPKαT176D overexpression reduces *Plasmodium falciparum* infection prevalence and intensity. Transgenic (hemizygous and homozygous) mosquitoes and wild type controls were provided with an artificial bloodmeal enriched with *P. falciparum* NF54 gametocytes. Ten days after infection, midguts were dissected and the numbers of *P. falciparum* oocysts were counted. (**A**) Prevalence reflects the percentage of mosquitoes infected with at least one oocyst in the midgut. (**B**) Intensity of infection reflects the mean number of oocysts found in infected mosquitoes. The mean numbers of oocysts per midgut ± SEMs are plotted. WT *n* = 91; homozygous TG *n* = 94; hemizygous TG *n* = 87. Infection prevalence was analyzed by Fisher’s exact test and intensity by Mann–Whitney (** *p* < 0.01, *** *p* < 0.001).

Increased AMPK activity in the midgut epithelium led to a significant decrease in the percentage of homozygous TG *An. stephensi* infected with *P. falciparum* (prevalence) and the number of oocysts in the midguts of infected mosquitoes (intensity; Figure 7). Specifically, the prevalence of infection was reduced from 51% in wild type to 27% in homozygous TG *An. stephensi* (*p* = 0.001, Figure 7A). The intensity of infection in homozygous TG mosquitoes was 0.37 oocysts/midgut (*n* = 94) compared to 0.79 oocysts/midgut in wild type *An. stephensi* (*n* = 90, *p* < 0.001; Figure 7B), suggesting that increased AMPK activity in the mosquito midgut can enhance resistance to *P. falciparum* infection. In contrast, for hemizygous TG *An. stephensi* there were no differences in prevalence (*p* = 0.11), which was intermediate to that of wild type and homozygous TG prevalence, or intensity of infection (*p* = 0.24) relative to wild type control (Figure 7A,B).

## 4. Discussion

AMPK is present in cells of all multicellular organisms and influences diverse physiological functions through its role in metabolic regulation [52,53]. In response to low energy levels, AMPK inhibits anabolic processes and activates catabolic processes [1]. In this study, we overexpressed a truncated and constitutively activated AMPK α-subunit in the *An. stephensi* midgut. As expected, we observed conserved effects on key metabolites in mosquitoes, including glycogen and trehalose, concurrent with increased HA-AMPKαT176D midgut expression. Further, HA-AMPKαT176D expression was not associated with any alteration in lifespan in hemizygous or homozygous female TG *An. stephensi*, but it was associated with decreased lifetime fecundity and decreased egg production in individual homozygous TG females relative to wild type controls. Finally, we observed that midgut HA-AMPKαT176D overexpression enhanced resistance to *P. falciparum* infection as indicated by decreased infection prevalence and intensity in homozygous TG mosquitoes compared to wild type controls.

The association between HA-AMPKαT176D midgut overexpression and reduced development of *P. falciparum* in our studies is consistent with reports of AMPK regulation of both exoerythrocytic and erythrocytic malaria parasite development [54,55], suggesting that host AMPK activity regulates *Plasmodium* development and growth in both mammals and vector mosquitoes. Specifically, overexpression of a constitutively active AMPK in human hepatic cells decreased growth of the mouse parasite *Plasmodium berghei* in vitro as evidenced by smaller hepatic schizonts compared to controls [55]. Furthermore, treatment of hepatocytes infected with *P. berghei* with several AMPK activators (salicylate, metformin, 2-deoxy-D-glucose, and A769662) resulted in significantly decreased schizont size, while salicylate treatment cells infected with *Plasmodium yoelii* or *P. falciparum* yielded similar results [55]. Provisioning of mice with the AMPK agonist metformin was also associated with decreased *P. yoelii* parasitemia [54]. Although the mechanism(s) by which increased AMPK activity impairs *Plasmodium* exoerythrocytic, erythrocytic and sporogonic development are not yet clear, we can speculate on several possible mechanisms. AMPK activation inhibits acetyl CoA carboxylase, crucial in fatty acid synthesis, resulting in decreased levels in cellular lipids [56]. Malaria parasites cannot synthesize sterols and are dependent on lipids from the host [57,58], so host AMPK activation could deprive parasites of essential sterols such as cholesterols and negatively impact parasite development. However, we did not observe significant changes in lipids in our transgenic mosquito line and thus this is likely not the case in these studies. Alternatively, a previous study found that *P. berghei* infection of *An. stephensi* was associated with enhanced glycogen accumulation in the mosquito host; the authors proposed that this metabolic shift promoted survival of infected mosquitoes and, therefore, of the parasites in these mosquitoes [59]. Based on these findings and our observations of decreased glycogen content in the absence of any observable changes in *An. stephensi* lifespan in both TG and AICAR-treated mosquitoes, AMPK activation could deprive developing parasites of other nutrients, perhaps related to the reduction in host glycogen. Finally, AMPK directly influences mitochondrial homeostasis and we have previously demonstrated that mitochondrial dysfunction in the mosquito midgut can profoundly affect *P. falciparum* development [31].

Consumption of the bloodmeal initiates a reproductive cycle in female mosquitoes, with proteins and lipids from the blood being used for both self-maintenance and reproduction. In *Ae. aegypti*, the bloodmeal is rapidly processed with approximately 10% of the nutrients devoted to egg production, 10% to somatic maintenance and the remainder removed as CO_2_ and waste [40]. Provisioning of AMPK activating agents have been reported to cause a delay of follicle development in *Gc. atropalpus*, irrespective of nutrient status [21]. Our data substantially extend these findings by specifically targeting overexpression of HA-AMPKαT176D in the *An. stephensi* midgut. With overexpression, we noted significantly decreased egg production in homozygous TG mosquitoes at the population level and in individual mosquitoes relative to controls during the first two reproductive cycles. Increased AMPK activation signals an energy deprived state, which likely induces a temporary switch in resource allocation away from egg development and towards somatic maintenance. Other studies in *C. elegans* and vertebrates have reported negative effects of AMPK activation on reproduction or reproductive cells [11,18,19,60,61,62]. These studies will inform our future efforts to identify the mechanism(s) of AMPK regulation of reproduction in *An. stephensi*.

In contrast to many studies conducted in other organisms [9,12,14,63,64], we did not observe an increase in lifespan with midgut overexpression of HA-AMPKαT176D in either the hemizygous or homozygous TG lines. Initially, we hypothesized that the lack of a nuclear transport signal might trap the transgene protein in the nucleus, limiting its biological activity as reported in an earlier study [48]. However, this was not the case, as the transgene protein was found at comparable levels in both the nuclear and cytoplasmic fractions. Our observations are consistent with a previous study that showed localization of a truncated AMPKα across cytoplasmic, mitochondrial, and nuclear fractions, with comparable phosphorylation of specific substrates in these different subcellular compartments [65]. Other studies have examined the impact of AMPK activity on lifespan by manipulating AMPK activity globally across tissues, either through pharmacological activators or genetic mutations [8,9,10,11]. Midgut-specific expression of HA-AMPKαT176D, however, is unlikely to be the reason for lack of an effect on lifespan because midgut AMPK activity has been directly connected to lifespan regulation in *D. melanogaster* [14]. A more likely explanation is the lack of sustained effects of AMPK overexpression in the *An. stephensi* midgut. The carboxypeptidase promoter we used to drive midgut-specific expression is leaky in *An. stephensi*, leading to consistent low level transgene expression in the midgut [29,32,66]. Despite this, we observed increased carboxypeptidase promoter activity from 6–24 h post-BM with a corresponding increase in transgene expression. As demonstrated with our metabolic assays, homozygous TG mosquitoes exhibited changes in nutrient metabolism, but only for a short period of time at the peak of transgene protein expression (i.e., 24 h). These transient changes may not have been sufficient to alter the survival of adult mosquitoes. Interestingly, lifespan was also not extended in *D. melanogaster* provisioned with the AMPK activator metformin at concentrations that were not toxic, even though increased AMPK activity was observed [15]. Additional studies with a more robust promoter for sustained transgene expression are likely going to be necessary to define the impacts of AMPK activity on mosquito lifespan.

In summary, our study provides the first evidence that overexpression of a constitutively activated form of AMPK in the mosquito midgut can impact *P. falciparum* invasion and development in *An. stephensi*. We verified that the core function of AMPK—regulation of host metabolism—is fully functional in anopheline mosquitoes and that this manipulation is sufficient to alter nutrient stores in the mosquito. Furthermore, targeting AMPK activity to the midgut restricted the allocation of resources to reproduction, resulting in significantly reduced fecundity. Together these data suggest that AMPK could be an interesting target for manipulating life history traits and infection competence in critically important malaria vector mosquitoes to reduce the burden of disease.

## Data Availability

The data presented in this study are available on request from the corresponding author.

## Supplementary Materials

The following are available online at https://www.mdpi.com/2073-4425/12/1/119/s1. Figure S1: A. Site-directed mutagenesis of Thr-176 of AMPKα protein. (A) Sense and antisense primers (A and B) span the start and stop codon, while internal sense and antisense primers encode the mutation. The first amplification involves two separate reactions (1a and 1b) using primers A and B & primers C and D respectively. The second amplification step (2) used primers A and D to amplify the full-length AMPKαT176D (3) using both amplicons from reaction 1a and 1b as templates. (B) Schematic of the inverse PCR product sequence. Genomic DNA from He-AMPKαT176D mosquitoes was cut with Mbol and was self-ligated to form circularized DNA, which was then used as a template for PCR with pBac-specific primers (Table S1. The amplified product from the putative insertion site was flanked with known pBac sequence and included 118 bp of unique genomic DNA sequence. The transgene was inserted into a TTAA sequence, the preferred site of pBac transposition, and did not disrupt any known or predicted *A. stephensi* genes. Figure S2: Effect of HA-AMPKαT176D in the midgut on nutrient stores, glycogen, lipids, and trehalose in hemizygous TG *An. stephensi* mosquito (He-AMPKαT176D). (A) Glycogen (B) trehalose, and (C) lipids were extracted and quantified from nine independent cohorts of female mosquitoes. Average titers and the SEM are shown No significant differences between NTG and He-AMPKαT176D mosquitoes were observed using the Student *t* test. Figure S3: Effect of the AMPK activator, AICAR, on nutrient stores in adult female *An. stephensi.* (A) Glycogen (B) trehalose, and (C) lipids were extracted and assayed from 16 biological replicates of pooled females (*n* = 5 females/pool). Average titers and SEM are shown; (* *p* < 0.05 per Student *t* test). Figure S4: Survivorship and mortality curves for sugar-fed and blood-fed hemizygous TG and NTG *An. stephensi*. Two sugar-fed and five blood-fed replicate survival studies were performed using TG and NTG siblings reared under identical conditions. Bloodmeals were provided daily to induce expression of HA-AMPKT176D transgene. Sucrose (10%) was provided *ad libitum* for all treatments. Survival curves were analyzed using the Kaplan-Meier method and significant differences were detected using the Wilcoxon test. Figure S5: Survivorship and mortality curves for sugarfed and blood-fed homozygous TG and wild type *An. stephensi*. Five sugar-fed and two blood-fed replicate survival studies were performed using Ho-TG and WT siblings reared under identical conditions. Bloodmeals were provided daily to induce expression of HA-AMPKT176D transgene. Sucrose (10%) was provided *ad libitum* for all treatments. Survival curves were analyzed using the Kaplan-Meier method and significant differences were detected using the Wilcoxon test. Figure S6: Effect of AICAR on wild type *An. stephensi* fitness. (A) A representative survivorship curve comparing wild type *An. stephensi* females maintained on a 10% sucrose solution with either 2mM AICAR or 1% PBS. Both groups were also provided a daily bloodmeal. (B) Lifetime fecundity of wild type *An. stephensi* females provisioned with AICAR or PBS throughout their lifespans. C. Cumulative egg counts per individual mosquito after each reproductive cycle. Figure S7: Cumulative egg production of *An. stephensi* per individual mosquito in TG mosquitoes across replicates (A)–(C). Total number of eggs per individual mosquito after each reproductive cycle in hemizygous TG female mosquitoes and NTG sibling controls. (D)–(E). Total number of eggs per individual mosquito after each reproductive cycle in homozygous TG female mosquitoes and wild type controls. Table S1: Sequences of primers used in this work.
