# Supplementary figures and images for: Overexpression of Activated AMPK in the Anopheles stephensi Midgut Impacts Mosquito Metabolism, Reproduction and Plasmodium Resistance
Source: Genes (Basel). 2021 Jan 19;12(1):119. doi: 10.3390/genes12010119 (PMC7835765; doi:10.3390/genes12010119)

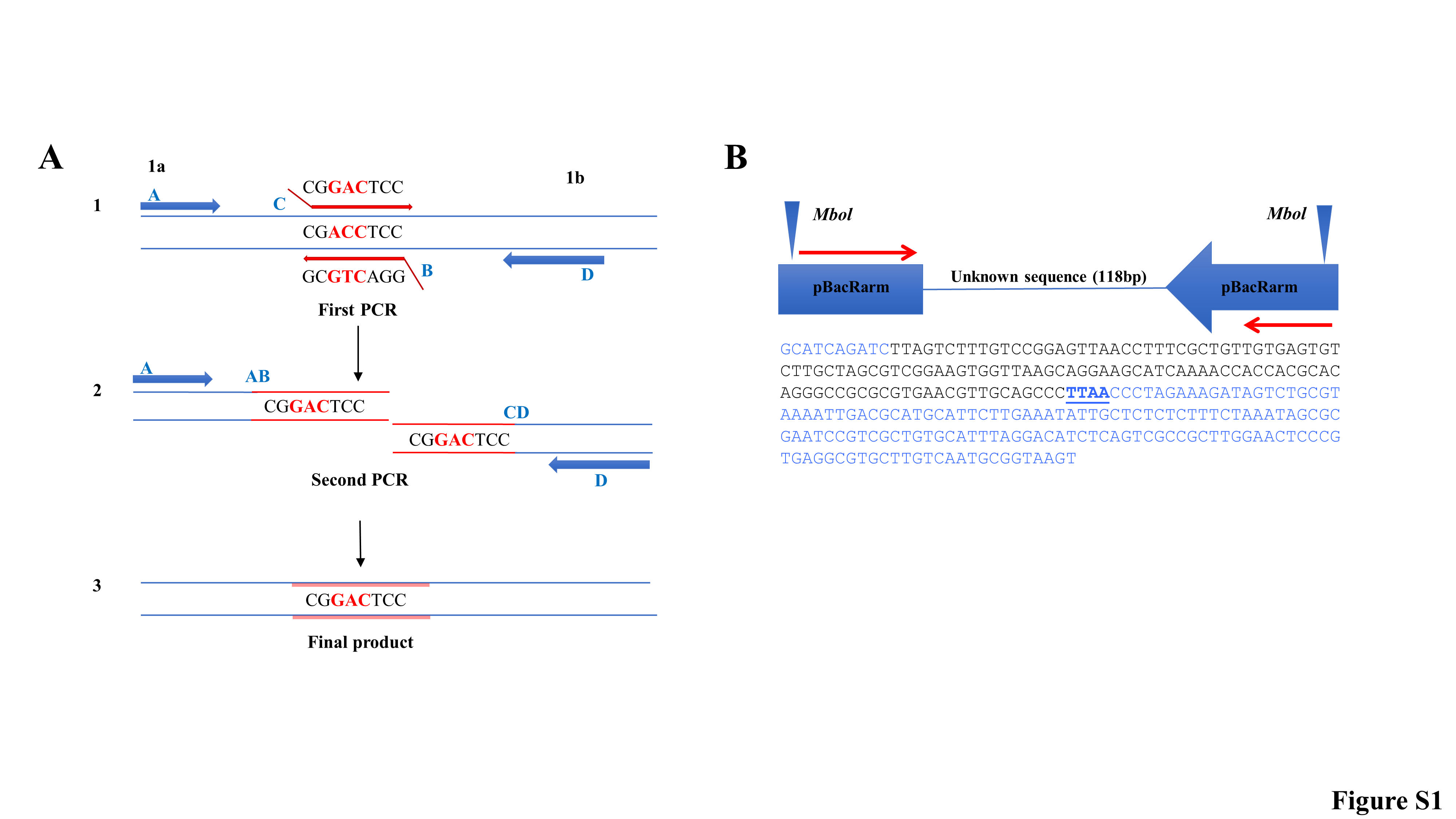

Supplement: Supplementary file 1 [file genes-12-00119-s001.zip › Figure S1.TIF]

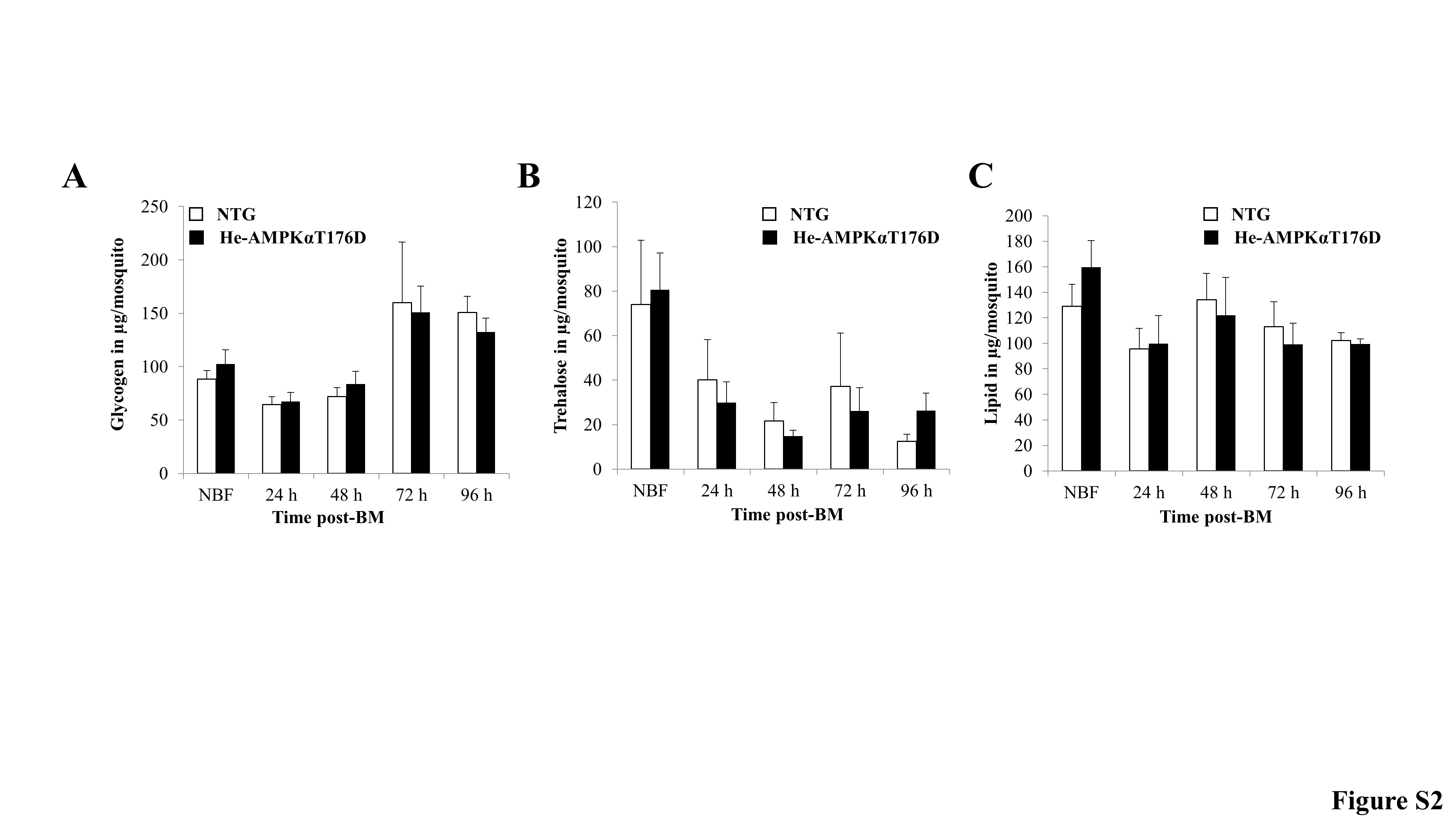

Supplement: Supplementary file 1 [file genes-12-00119-s001.zip › Figure S2.TIF]

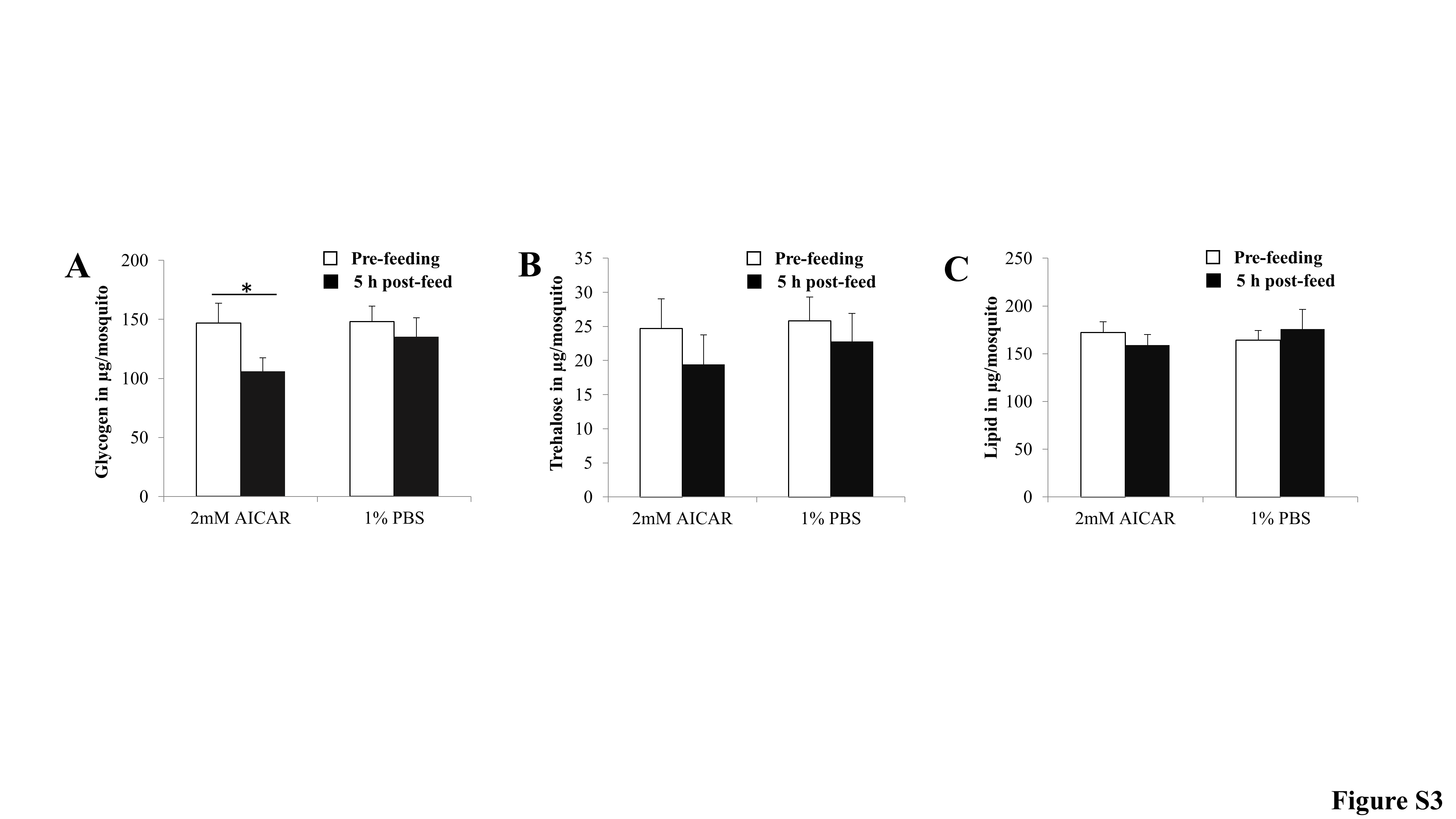

Supplement: Supplementary file 1 [file genes-12-00119-s001.zip › Figure S3.TIF]

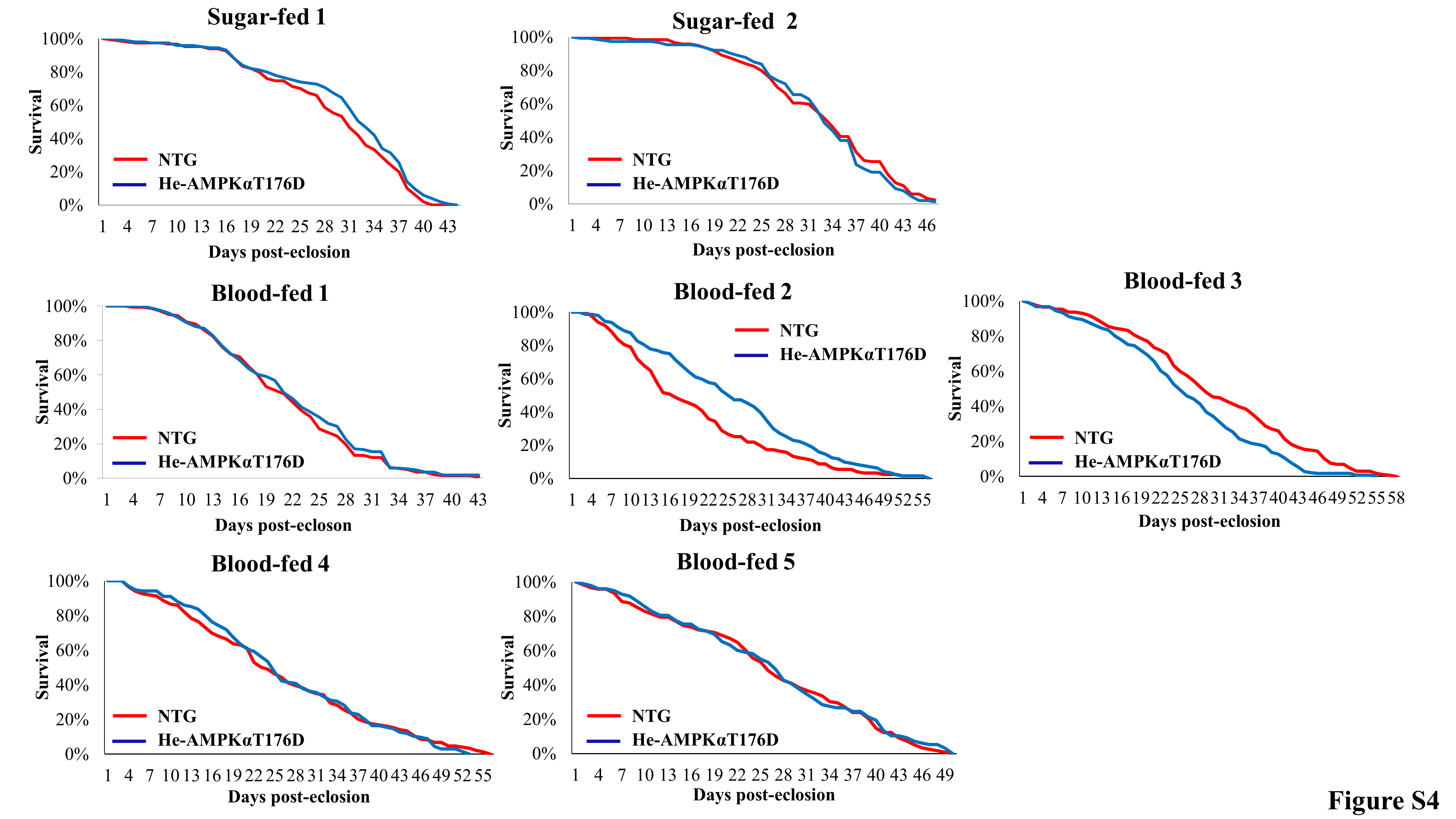

Supplement: Supplementary file 1 [file genes-12-00119-s001.zip › Figure S4.TIF]

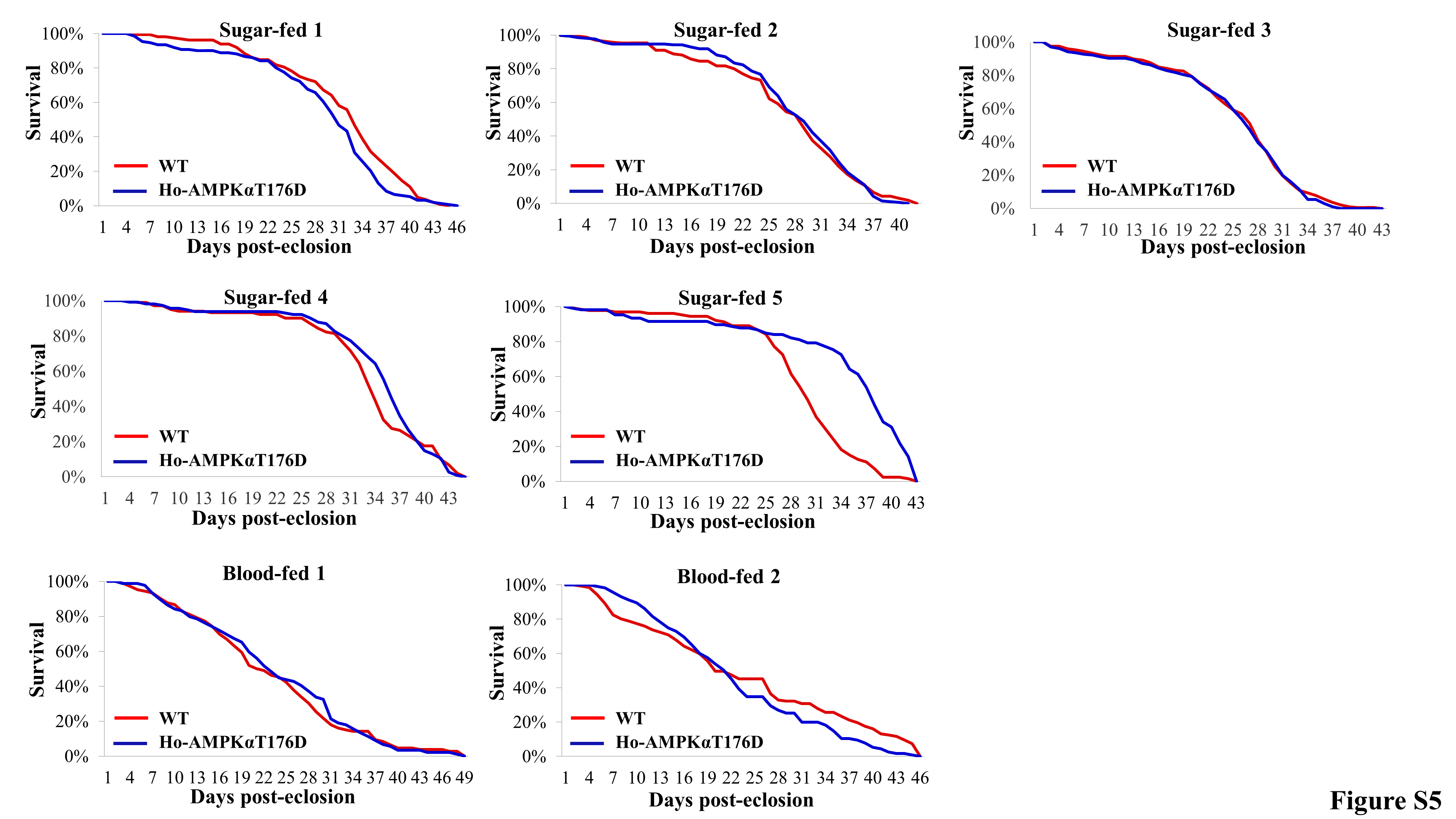

Supplement: Supplementary file 1 [file genes-12-00119-s001.zip › Figure S5.TIF]

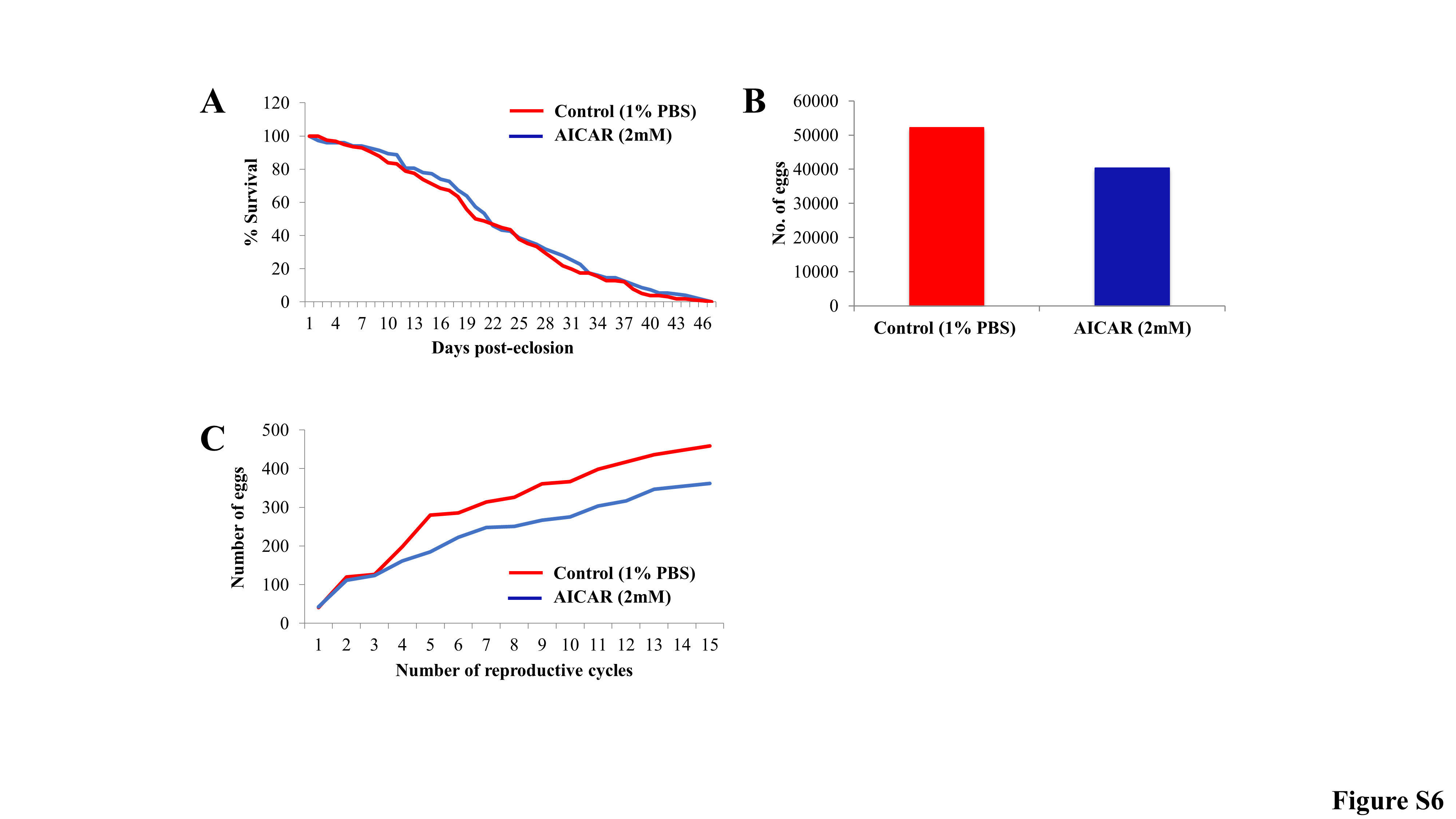

Supplement: Supplementary file 1 [file genes-12-00119-s001.zip › Figure S6.TIF]

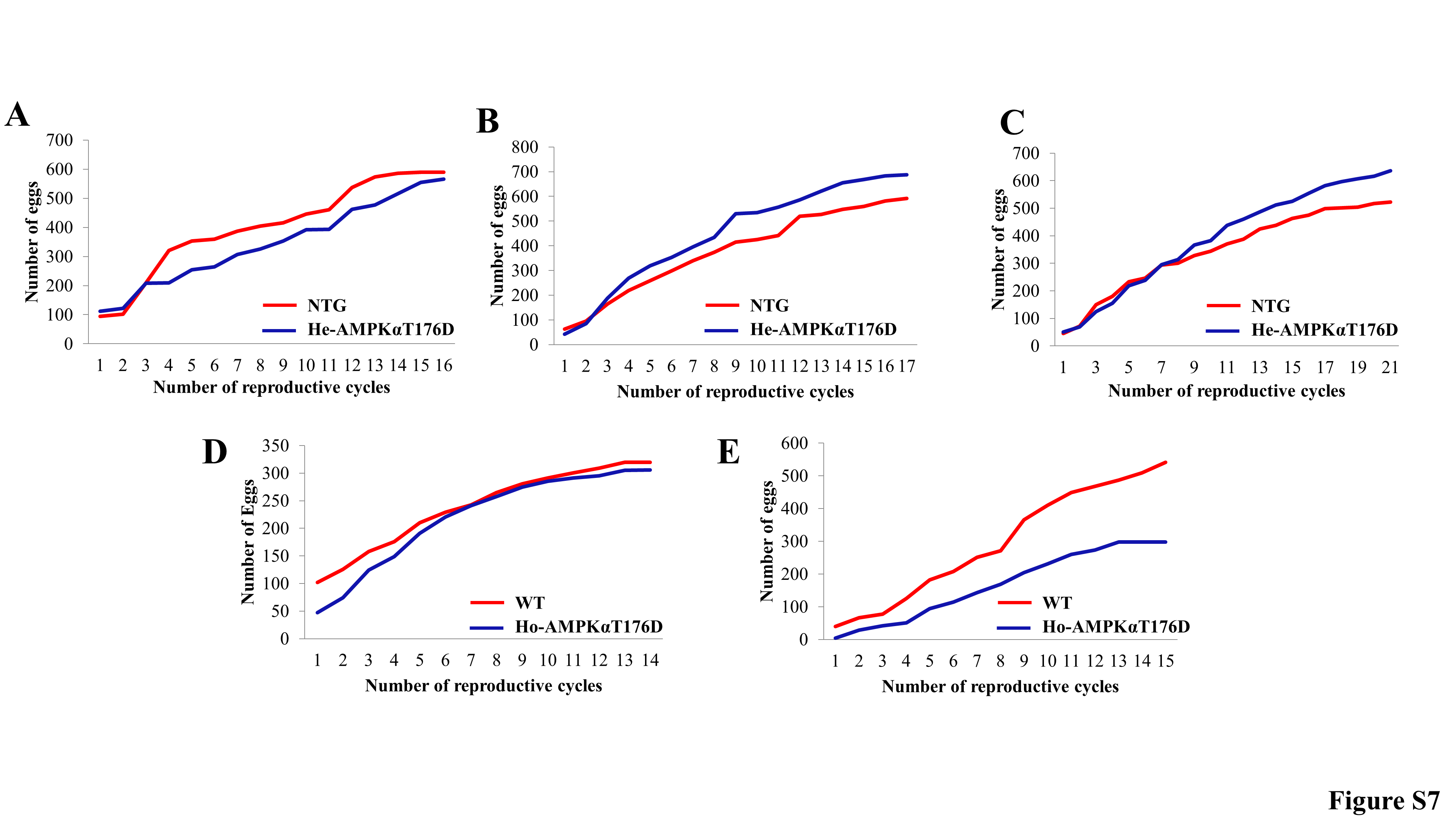

Supplement: Supplementary file 1 [file genes-12-00119-s001.zip › Figure S7.TIF]

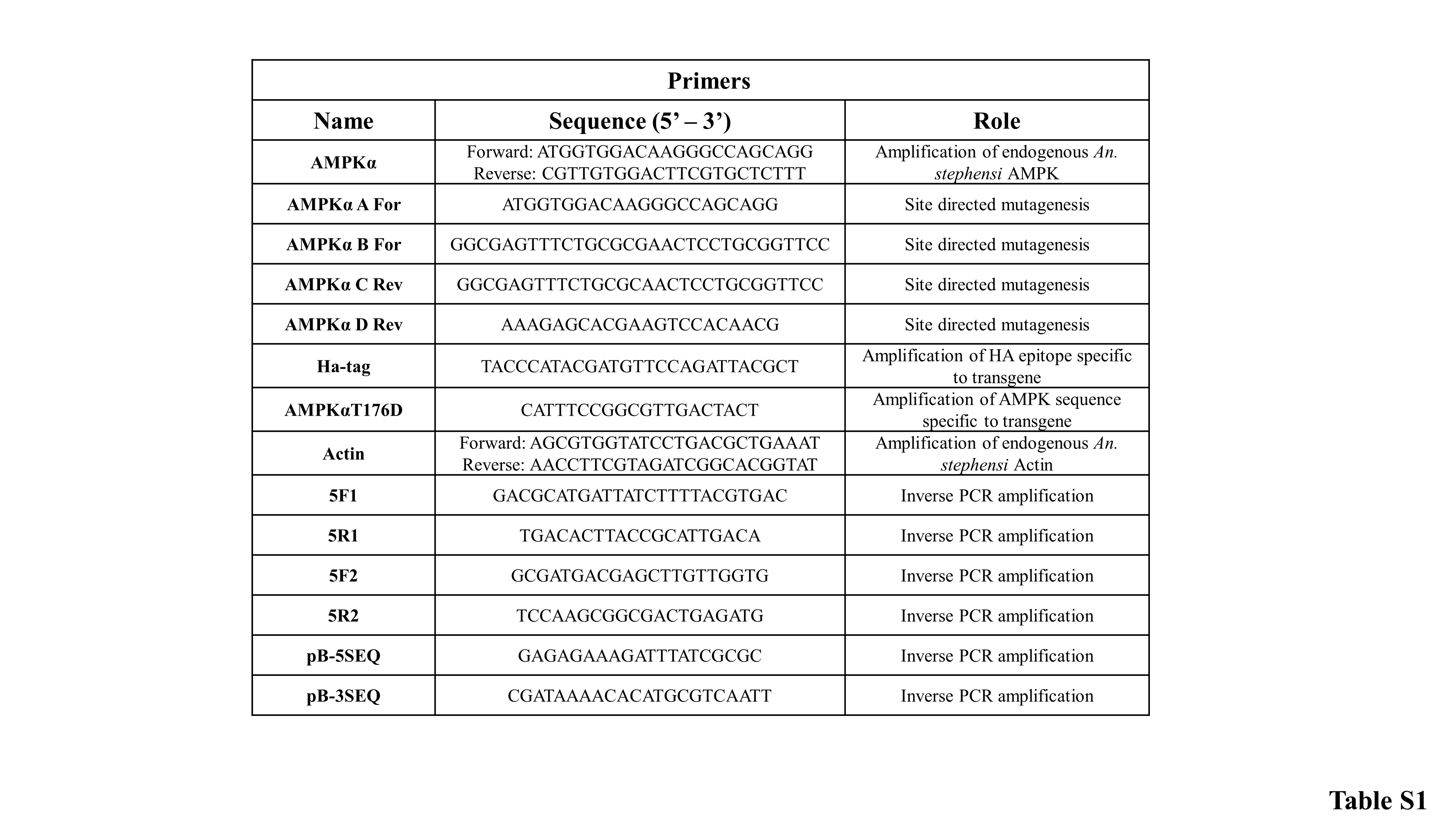

Supplement: Supplementary file 1 [file genes-12-00119-s001.zip › Table S1.TIF]
